# The complete chloroplast genome sequence of *Dactylorhiza majalis* (Rchb.) P.F. Hunt et Summerh. (*Orchidaceae*)

**DOI:** 10.1080/23802359.2019.1660282

**Published:** 2019-09-02

**Authors:** Michał May, Alžběta Novotná, Julita Minasiewicz, Marc-Andre Selosse, Marcin Jąkalski

**Affiliations:** a Department of Plant Taxonomy and Nature Conservation, Faculty of Biology, University of Gdańsk, Gdańsk, Poland;; b Faculty of Science, University of South Bohemia, České Budějovice, Czech Republic;; c Institut de Systématique, Evolution, Biodiversité (ISYEB), Muséum national d'Histoire naturelle, CNRS, Sorbonne Université, EPHE, Paris, France

**Keywords:** Chloroplast genome, *Dactylorhiza majalis*, orchid, *Orchidaceae*, next-generation sequencing

## Abstract

The complete chloroplast genome of *Dactylorhiza majalis* (Rchb.) P.F. Hunt et Summerh. (*Orchidaceae*:*Orchidoideae*) was assembled and characterized using next-generation sequencing data. The plastome (154,108 bp) possesses the typical circular structure consisting of a large single-copy region (LSC; 83,196 bp), a small single-copy region (SSC; 26,580 bp), and two copies of inverted repeats (17,752 bp each). Its overall GC content is 36.99% and the plastome encodes 134 genes. Reconstruction of phylogenetic relationships using complete plastome sequences of *Orchidaceae* representatives showed that *D*. *majalis* was nested within the *Orchidoideae* tribe *Orchideae*. The complete plastome comprises a valuable tool in elucidating taxonomic uncertainties within the genus *Dactylorhiza*.

*Dactylorhiza* Necker *ex* Nevski is a temperate orchid genus known from its complex evolutionary relationships between species driven by high frequency of hybridization, introgression, and polyploidization. Reticulate evolution pattern along with relatively great morphological variability within a species is considered a major challenge in taxonomy of the genus (Hedrén 1996; Pillon et al. 2007, and references therein). *Dactylorhiza majalis* (Rchb.) P.F. Hunt et Summerh. can be found in western and central Europe, Baltic region, and northern Russia (Hultén and Fries 1986; Balao et al. 2016). It is an allotetraploid species belonging to a polyploid complex formed iteratively by crosses between *Dactylorhiza incarnata* s.l. and *Dactylorhiza maculata* s.l. with the last species being always maternal parent (Hedrén et al. 2008 and references therein). Complete, annotated plastidial genome, upon which new molecular markers can be described, would be a valuable tool in untangling evolutionary history within the genus. Chloroplast genomes provide researchers with data invaluable for resolving major phylogenetic relationships between orchid subfamilies (Givnish et al. 2015). Currently, only 15 complete chloroplast genomes are available within the subfamily *Orchidoideae* (Delannoy et al. 2011; Lin et al. 2015; Yu et al. 2015; Zhu et al. 2016; Roma et al. 2018; Lallemand et al. 2019; Oh et al. 2019). This makes this subfamily largely underrepresented among other orchids. Species from the *Dactylorhiza* genus were so far only subject to phylogenetic studies employing ITS, microsatellite loci, selected marker genes, or morphology, and results of these still often remain incongruent (Bateman et al. 2003; Shipunov et al. 2004; Balao et al. 2016; Jin et al. 2017).

Fresh leaves were collected from an individual growing in Psary, Poland (N50°22′07.4″ E19°04′53.3″). Leaves dried in silica gel (voucher SG-13237, Herbarium of University of Gdansk, UGDA) were used for extraction of the total genomic DNA with Dneasy Plant Mini Kit (Qiagen, Hilden, Germany). Sequencing library was generated with Accel-NGS^®^ 1S Plus DNA Library Kit (Swift Biosciences Inc., Ann Arbor, MI). Next-generation paired-end sequencing was performed with Illumina HiSeq 4000 (San Diego, CA). The obtained reads were used for genome assembly with the Geneious software version 10.2.4 (https://www.geneious.com) with medium-low sensitivity parameters and a subset of 25% of the reads, followed by mapping to the closest reference plastome (*Platanthera japonica*, NC_037440.1), and reassembly with medium sensitivity parameters to increase the assembly quality. Annotation was performed within Geneious as well as using GeSeq (Tillich et al. 2017), and manually corrected afterwards. Phylogenetic relationships of *D. majalis* with other orchids were inferred from maximum-likelihood analyses with RAxML-NG (Kozlov et al. 2019) using selected available complete orchid plastomes aligned with MAFFT (Katoh and Standley 2013).

The chloroplast DNA of *D. majalis* is 154,108 bp in length, presenting the overall GC content (the proportion of guanine and cytosine bases) of 36.99%. Consistent with other known orchid plastomes it is comprised two inverted repeats (IRa and IRb) with 26,580 bp in length, an 83,196 bp large single-copy region (LSC), and a 17,752 bp long small single-copy region (SSC). A total of 134 genes were annotated, of which 113 are unique. These are 4 rRNA genes, 30 tRNA genes, and 79 protein-coding genes. Twenty genes are duplicated in the IR region. Additionally, 12 protein-coding genes and 6 tRNA genes contain introns. The annotated sequence was deposited at GenBank with the accession number MK984209. Results of the phylogenetic relationships investigation between *D. majalis* and other members of the *Orchidaceae* showed its clustering together with representatives of the subtribe *Orchidinae* (Figure 1). The complete plastome sequence we provided here constitutes a valuable aid for addressing the taxonomic uncertainties within the genus *Dactylorhiza*, as well as analysing the genetic diversity of the *Orchidaceae* family.

**Figure 1.**
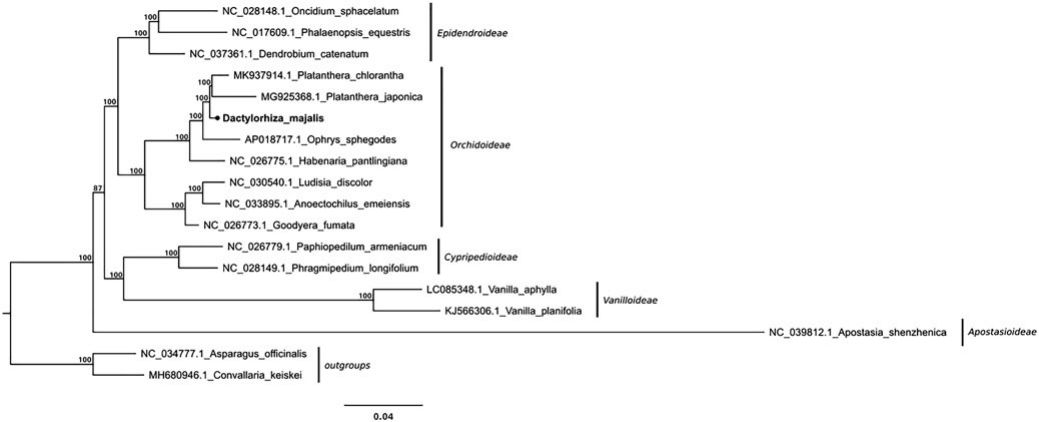
Phylogenetic relationships inferred from maximum-likelihood analyses with full length plastome sequences of *Orchidaceae* representatives, including the newly assembled *D. majalis* plastome. Node support values are derived from RAxML assessment with 1000 bootstraps replicates. Non-orchid monocots were used for tree rooting.
